# PET Response-Guided Treatment of Hodgkin's Lymphoma: A Review of the Evidence and Active Clinical Trials

**DOI:** 10.1155/2011/309237

**Published:** 2010-12-27

**Authors:** Paul Aridgides, Jeffrey Bogart, Anna Shapiro, Ajeet Gajra

**Affiliations:** ^1^Department of Radiation Oncology, SUNY Upstate Medical University, Syracuse, NY 13210, USA; ^2^Division of Hematology-Oncology, Department of Internal Medicine, SUNY Upstate Medical University, Syracuse, NY 13210, USA

## Abstract

Risk-adaptive therapy for Hodgkin's lymphoma focuses on treatment modifications based on assessment of response. [^18^F]Fluoro-deoxyglucose positron emission tomography (PET) performed during or after completion of chemotherapy is a strong prognostic factor for eventual treatment outcome. Conceptually, this strategy seeks to increase efficacy and minimize toxicity through the appropriate selection of patients for either therapy escalation (high-risk, PET positive) or de-escalation (low-risk, PET negative). Preliminary evidence with tailoring both chemotherapy (drug selection, number of cycles, and dose) and radiotherapy (omission or inclusion) is varied; however, numerous clinical trials seeking to validate this approach are ongoing. This paper summarizes the available evidence and active protocols involving PET response-adapted therapy for adult (early and advanced stages) Hodgkin's lymphoma.

## 1. Introduction

Treatment of Hodgkin's lymphoma (HL) with modern combination chemotherapy and radiation therapy has increased long-term survival to greater than >75% [1]. In the United States, most patients receive a chemotherapy backbone consisting of ABVD (doxorubicin, bleomycin, vinblastine, dacarbazine) with or without consolidative involved-field radiotherapy (IFRT) [2]. Efforts to improve outcomes in advanced-stage disease have lead to development of intensified chemotherapy regimens such as BEACOPP (bleomycin, etoposide, doxorubicin, cyclophosphamide, vincristine, procarbazine, prednisone), but there is concern for increased risk of hematologic side effects and acute leukemia [3]. Consolidative IFRT following chemotherapy for advanced stage disease may be included as part of certain treatment regimens [4, 5]. Stanford V (abbreviated, dose-intense) chemotherapy with IFRT has yielded promising results in a phase II trial, with results from an intergroup trial (ECOG-2496) comparing Stanford V and ABVD awaited [6]. 

In an effort to tailor treatment towards both favorable and unfavorable-risk patients, response-adaptive therapy involves modifications (de-escalation or intensification, resp.) based upon response assessment. The prognostic value of PET, both during and after completion of treatment, has been demonstrated [7]. As a result, there has been active interest in developing protocols which combine conventional stratification (stage, bulky disease, adverse prognostic factors) with assessment of PET response. This review will focus on describing both the preliminary evidence regarding PET-adaptive therapy as well as the mechanisms (chemotherapy and radiotherapy modifications) being investigated in ongoing clinical trials.

## 2. Prognostic Value of Early Interim PET Response

Studies in high-grade non-Hodgkin's lymphoma, with the inclusion of a few HL patients, first demonstrated a strong predictive value of PET scan performed after 1-2 cycles of chemotherapy [8–10]. In a subsequent analysis of 85 patients with HL, Hutchings identified PET response after two or three cycles of chemotherapy to be an accurate and independent predictor of progression-free survival (PFS) and overall survival (OS). All patients with interim PET-positive scans suffered disease relapse within 2 years. Furthermore, patients classified at the time as having “minimal residual uptake” on PET behaved similarly to PET-negative patients, suggesting the consideration of this finding as a PET complete response (CR) [11].

In a Danish prospective analysis, 77 patients with HL of all stages (63% Stage I/II) were treated with ABVD chemotherapy and IFRT as per institutional protocols. PET was performed after two cycles (PET-2) of chemotherapy; however, no treatment modification was made based on PET response. With 11 out of 16 (69%) PET-2-positive patients relapsing or progressing, compared to just 3 out of 61 (5%) PET-2-negative patients, PET-2 response was found to be a significant predictor for both PFS and OS (*P* < .01). PET-2 response was the strongest predictor of PFS on multivariate analysis, and PET was proved superior to conventional computer tomography (CT) imaging [12]. An Italian prospective trial examined PET-2 response in 108 patients (mostly advanced stage) treated with ABVD with 54% receiving IFRT. PET-2 response correctly predicted treatment outcome in 95% of patients, with a positive predictive value of 90% and a negative predictive value of 97% [13]. 

 In a combined analysis of the Italian and Danish prospective trials, the 2-year PFS was 12.8% for PET-2-positive patients compared to 95.0% if PET-2 negative (*P* < .001). PET-2 response was the only significant predictor of outcome on multivariate analysis (*P* < .0001). Both the negative predictive value (NPV) and positive predictive value (PPV) of PET-2 response were excellent (92% and 93%, resp.) [14]. In a systematic review involving 360 patients with advanced-stage HL, interim PET/CT had an overall sensitivity of 81% and specificity of 97% [15]. It should be emphasized that this analysis reflects the prognostic value of interim PET in patients continuing with planned treatment regimens. These numerical values cannot be extrapolated to predict outcomes if modifications are made, but do indicate an overall good outcome following treatment in patient with interim PET-CR.

## 3. Chemotherapy Modification Based on PET Response

One approach under investigation for early-interim PET-negative patients, who have an improved prognosis, is to employ chemotherapy reduction strategies. In advanced-stage disease, where higher doses of chemotherapy are indicated, omitting chemotherapy cycles or changing to less-intensive chemotherapy could potentially spare treatment-related toxicity with the goal of maintaining efficacy. The major risk of treatment de-escalation, even following an interim good response, is the potential for inferior outcomes as a result of less-intensive therapy. In contrast for patients found to be interim PET-positive, this may indicate aggressive disease for which chemotherapy escalation would be beneficial. The early experience from two Israeli trials employing chemotherapy modification is reviewed below. 

Investigators in Haifa, Israel, prospectively treated 108 HL patients (all stages) with an initial two cycles of escalated BEACOPP (BEACOPPesc) or standard dose BEACOPP chemotherapy based on pretreatment risk stratification. Subsequent therapy was based on PET-2 response, with PET-2-negative patients receiving standard BEACOPP and PET-2-positive patients receiving BEACOPPesc (both 4 cycles). Protocol indications for radiation therapy, given to 36% of patients, included initial bulky disease (>10 cm) and a single PET-positive site after completing chemotherapy. Of the 39 patients with initial high-risk disease (international prognostic score, IPS > 3), 79% were PET-2 negative after two cycles of escalated BEACOPP and subsequently received 4 cycles of standard dose BEACOPP. Interim PET-based treatment was effective and feasible, with 5-year event-free survival (EFS) and OS of 85% and 90%, respectively [16]. 

In a phase II study of 43 patients, investigators at Hadassah University Hospital, Jerusalem, treated advanced stage HL (IPS > 3, bulky IIB, III, and IV) with 2 cycles of escalated BEACOPP and then evaluated for PET response [17]. Patients with a favorable PET response received an additional 4 cycles of ABVD. Results of this study were comparable to similar patients (high-risk, advanced HL) treated in the German HD9 trial with 8 cycles BEACOPPesc. Additionally, PET response was prognostic for treatment outcome; the 4-year PFS for PET-2-negative patients was 87%, compared to 53% if PET-2 positive. The results of these single-institution studies, involving relatively few patients, do suggest feasibility with chemotherapy modification based on interim PET response. Applicability with regard to less intensive ABVD chemotherapy, as compared to the BEACOPP-based regimens employed, is unknown. These data require validation in large clinical trials, which are underway, before incorporation into general practice.

## 4. Consolidative Radiation Therapy Based on PET Response

There remains continued interest in identifying which patients may benefit from consolidative IFRT following systemic chemotherapy. PET response has been investigated in this regard as well. As with chemotherapy treatment modification, there is potential for over-treatment (PET false positive) as well as the risk of inferior outcome with omission of IFRT (PET false negative). Early studies utilizing this approach, summarized below, are varied with regard to outcomes, early-versus late-stage patients, and chemotherapy regimens employed. 

In an analysis of 81 patients (72% early stage) treated with Stanford V chemotherapy and IFRT, PET positive patients after completion of chemotherapy did significantly worse than PET negative patients (FFP 33% versus FFP 96%, resp. *P* < .0003). IFRT (20 to 30 Gy) was given to all patients and sites of relapse included fields treated by radiotherapy for all patients with recurrence. Thus, in patients with PET-positive disease after Stanford V chemotherapy, treatment with low-dose IFRT is likely inadequate [18]. In contrast, researchers at Dana Farber Cancer Institute supported the use of consolidative IFRT in PET-positive patients after completion of 4 to 6 cycles of ABVD. While PET-positive patients after chemotherapy did worse (2-year FFS 69% compared to 95% PET negative, *P* < .01), the authors conclude that IFRT potentially cured a proportion of patients with persistent PET-positive disease following ABVD [19]. Conclusions from this study are limited due to its retrospective nature, and therefore the effect of low-dose IFRT following ABVD (for both PET-positive and PET-negative patients) is difficult to ascertain. 

Within the HD15 trial of the German Hodgkin Study Group (GHSG), 817 patients (stage IIB bulky/extranodal, III, IV) were randomized to three variations of BEACOPP chemotherapy and assessed for response by PET-CT at completion (6 to 8 cycles). Patients with PET-positive residual disease (>2.5 cm in size) received 30 Gy IFRT. The PFS for patients with PET-negative residues (treated with chemotherapy alone) was 96% compared to 86% for PET-positive patients treated with chemotherapy and IFRT (*P* = .011). While these data are preliminary, patients with a PET-CR after 6–8 cycles of BEACOPP chemotherapy were spared radiotherapy with no impact on early progression or relapse [20]. In the only published randomization evaluating IFRT based on PET response, investigators in Naples, Italy, treated 260 patients with bulky HL (>5 cm, all stages) with VE-BEP (vinblastine, etoposide, bleomycin, epirubicin, prednisone) chemotherapy for six cycles and assessed PET response. Patients with a PET CR after completion of chemotherapy were randomized to treatment with or without IFRT (32 Gy to initial bulky disease and contiguous nodal regions). Patients treated with chemotherapy alone, even after a PET CR, did worse (EFS 86% compared to 96% with IFRT, *P* = .03). Additionally, all relapses in patients treated without radiotherapy occurred in sites where IFRT would have been given. Of the patients with PET-positive disease after chemotherapy (treated with high-dose chemotherapy and stem cell transplant), 50% were alive and disease-free. The authors concluded that even after a PET CR, IFRT can improve EFS in patients with initial bulky disease [21]. 

Overall, the data regarding PET response-guided radiotherapy are conflicting, therefore it is too soon to draw reliable conclusions regarding the inclusion of radiotherapy based on PET CR. The most reliable data, from the GHSG HD15 trial, suggest that it is safe to omit RT following intensive BEACOPP chemotherapy (not commonly used in the US). Retrospective data confirm that with PET-positive residues following chemotherapy (ABVD or Stanford V), these patients have a poor prognosis and therefore more aggressive disease. Further clinical trials should give insight to whether further chemotherapy, IFRT, or high-dose chemotherapy with stem cell rescue should be employed in these patients. Additionally, no data exist regarding the impact of interim PET-2 response on the inclusion of IFRT and techniques employing these strategies are ongoing and summarized below.

## 5. Active Trials: Adult Early Stage HL

PET-based protocols for early-stage HL are currently evaluating the inclusion of IFRT only if PET-2 positive, as well as intensifying chemotherapy in these patients. CALGB trials 50604 and 50801 (non bulky and bulky Stage I/II, resp.) assess for PET response after an initial 2 cycles of ABVD (clinicaltrials.gov ID NCT01132807 and NCT01118026, resp.). Patients that are PET-2 negative receive ABVD alone (4 cycles if non bulky, 6 cycles if bulky), with radiotherapy omitted in these patients. PET-2-positive patients receive additional BEACOPPesc (2 cycles if nonbulky, 4 cycles if bulky) followed by consolidative IFRT (30.6 Gy to initial disease sites). The German HD16 for early-stage HL (favorable stage I/II) is testing 2 cycles of ABVD and 30 Gy IFRT compared to the same regimen with PET response-guided radiotherapy. Patients on the first arm receive IFRT regardless of PET-CT response, while patients on the second arm receive IFRT only if PET-2 positive (clinicaltrials.gov ID NCT00736320). The H10 EORTC/GELA study is recruiting patients with stage I/II disease to ABVD 3-4 cycles and 30 Gy INRT (involved node RT) versus PET-guided therapy. In the comparator arm, PET-2-negative patients receive an additional 2–4 courses ABVD only. PET-2-positive-patients receive BEACOPPesc for 2 cycles and 30 Gy INRT. As of July 2009, this trial had accrued 1,097 out of a planned 1,200 patients and the final results of this important trial is awaited [22].

## 6. Active Trials: Adult Advanced Stage HL

For advanced stage HL, current protocols focus on chemotherapy escalation or reduction based on PET-2-response. Trials from Cancer Research UK (stage IIB-IV) and Southwest Oncology Group (SWOG, stage III/IV) treat PET-2-positive patients after ABVD with BEACOPP-based regimens (NCT00678327 and NCT00822120, resp.). Chemotherapy randomizations following PET-2-positive disease compare BEACOPP-14 to BEACOPP-esc (SWOG) and BEACOPP to BEACOPPesc (Cancer Research UK). PET-2negative patients on the SWOG trial receive ABVD alone (4 additional cycles), whereas on the Cancer Research UK trial they are randomized to chemotherapy reduction (4 cycles ABV versus 4 cycles ABVD). 

To evaluate inclusion of radiotherapy based on PET response, an Italian protocol (stage III/IV) randomizes PET-2 negative patients to ABVD with or without IFRT (NCT00784537). PET-2 positive patients receive high dose chemotherapy and stem cell transplant. Based on favorable results in patients treated with chemotherapy alone after PET complete response on HD15, the German HD18 trial for advanced/unfavorable HL (stage IIB, III, IV) includes radiotherapy only for PET-positive resides (>2.5 cm) after completion of chemotherapy [clinicaltrials.gov ID NCT00515554]. This trial also tests randomizations for PET-2 negative patients receiving BEACOPPesc chemotherapy (4 versus 8 total cycles) and PET-2 positive patients (8 total cycles with or without rituximab).

## 7. Conclusions

PET response is strongly prognostic for treatment outcome in HL. As a result, interest has developed in tailoring treatment based upon PET assessment. While this approach has the potential to identify appropriate patients for either intensifying or deintensifying therapy, the mechanisms to do so remain largely undefined. Treatment de-escalation strategies carry a risk of negatively impacting the overall highly favorable outcomes in this disease. Likewise, there is no consensus regarding the appropriate treatment of PET-avid disease (interim or after completion of therapy), in which these patients may require intensive therapy. An active interest in PET response-based treatment strategies is demonstrated through widespread incorporation into Hodgkin's lymphoma protocols, but the variety of strategies being employed reflect a relative lack of experience with this approach. The results of these upcoming studies will hopefully answer the combined questions of whether and how treatment can be safely modified based on PET response.
